# Detection of Cracks on Tomatoes Using a Hyperspectral Near-Infrared Reflectance Imaging System

**DOI:** 10.3390/s141018837

**Published:** 2014-10-10

**Authors:** Hoonsoo Lee, Moon S. Kim, Danhee Jeong, Stephen R. Delwiche, Kuanglin Chao, Byoung-Kwan Cho

**Affiliations:** 1 Department of Biosystems Machinery Engineering, College of Agricultural and Life Science, Chungnam National University, 99 Daehak-ro, Yuseong-gu, Daejeon 305-764, Korea; E-Mail: Hoonsoolee83@gmail.com; 2 Environmental Microbiology and Food Safety Laboratory, Agricultural Research Service, U.S. Department of Agriculture, Powder Mill Rd. Bldg. 303, BARC-East, Beltsville, MD 20705, USA; E-Mails: moon.kim@ars.usda.gov (M.S.K.); kevin.chao@ars.usda.gov (K.C.); 3 Department of Food and Nutrition, Hanyang University, Seoul 133-791, Korea; E-Mail: jeongdanhee@gmail.com; 4 Fruit Quality Laboratory, USDA-ARS, Beltsville, MD 20705, USA; E-Mail: stephen.delwiche@ars.usda.gov

**Keywords:** hyperspectral near infrared reflectance imaging technique, crack tomato, imaging processing, principle component analysis, F-value

## Abstract

The objective of this study was to evaluate the use of hyperspectral near-infrared (NIR) reflectance imaging techniques for detecting cuticle cracks on tomatoes. A hyperspectral NIR reflectance imaging system that analyzed the spectral region of 1000–1700 nm was used to obtain hyperspectral reflectance images of 224 tomatoes: 112 with and 112 without cracks along the stem-scar region. The hyperspectral images were subjected to partial least square discriminant analysis (PLS-DA) to classify and detect cracks on the tomatoes. Two morphological features, roundness (*R*) and minimum-maximum distance (*D*), were calculated from the PLS-DA images to quantify the shape of the stem scar. Linear discriminant analysis (LDA) and a support vector machine (SVM) were then used to classify *R* and *D*. The results revealed 94.6% and 96.4% accuracy for classifications made using LDA and SVM, respectively, for tomatoes with and without crack defects. These data suggest that the hyperspectral near-infrared reflectance imaging system, in addition to traditional NIR spectroscopy-based methods, could potentially be used to detect crack defects on tomatoes and perform quality assessments.

## 1. Introduction

Tomatoes are the most popular vegetable by volume with world-wide production of approximately 162 million tons in 2012 [1]. They are considered to be healthy because they contain high levels of lycopene, which is a natural antioxidant, as well as β-carotene, vitamin C, and vitamin E [2]. However, raw tomatoes might contain sites that could harbor life-threatening pathogens. The United States Center for Disease Control (CDC) reported that tomatoes have been associated with 12 outbreaks of food poisoning in North America between 1990 and 2007 [3]. In 2000, a *Salmonellae javiana* infection occurred in 174 patients in four states after the consumption of raw tomatoes, and a *Shigella flexneri* outbreak affected 886 people in North America in 2001 [4]. Additional multistate outbreaks of *Salmonellae typhimurium* in the United States were associated with the consumption of raw tomatoes [5].

Cuticle cracks on tomatoes are created by changes in growth rate (e.g., excessively rapid fruit expansion due to high volumes of rainwater) and the splitting of the cuticle around the stem scar. It has been suggested that fecal matter, as well as contaminated soil and irrigation water, could infiltrate the fruit through cuticle cracks or the connecting tissue (stem-depression) between the stem and the fruit [6], allowing the growth and survival of pathogens, such as *Salmonella*. Therefore, it is essential to separate cracked from sound tomatoes for raw consumption.

Spectroscopic techniques are advantageous because they allow the simultaneous measurement of chemical and physical information from samples without causing destruction. Moreover, they can acquire both quantitative and qualitative information without the need for separate analyses. The near-infrared (NIR) spectral region has been widely used to assess the internal quality of fruits [7]. Specifically, NIR spectroscopy has been used to determine the content of soluble solids, lycopene, and β-carotene in tomatoes [8]. In addition, Xie *et al.* demonstrated that visible and NIR spectroscopy could be used to classify the different genotypes of tomatoes [9]. However, it is difficult to use conventional NIR spectroscopy to detect defects because the probed area is typically much smaller than the region of the tomato where defects commonly occur.

In recent years, hyperspectral imaging has been commonly employed to detect potential defects and contamination in vegetable and fruits. It combines the benefits of both machine vision and spectroscopy. Hyperspectral imaging systems can acquire hundreds of spectral readings of each spatial pixel in an image that contains thousands of such pixels; this information can then be used to assess the quality and safety attributes of foods [10,11]. A previous study by Kim *et al.* demonstrated that visible and NIR reflectance imaging could be used to detect defects and fecal matter on apples by performing measurements in the NIR region from 800 to 980 nm [12]. In addition, Guyer and Yang used hyperspectral imaging within the 680–1280 nm spectral range to detect defects in sweet cherries [13]. NIR hyperspectral reflectance imaging from 900 to 1700 nm was used to detect bruises on pickling cucumbers [14]. An advantage of NIR hyperspectral imaging is that can be used to perform multiple detection tasks; therefore, internal quality and surface defects could be assessed simultaneously using NIR hyperspectral imaging techniques.

The aim of this study was to evaluate the potential of hyperspectral NIR imaging in the spectral region from 1000 to 1700 nm to detect cracks on tomatoes. The specific objectives were to analyze the spectral response of tomatoes and to develop an optimal algorithm for detecting cracks using partial least square discriminant analysis (PLS-DA). Finally, geometric values were obtained from the resulting images, and linear discriminant analyses (LDA) and a support vector machine (SVM) were used to improve the accuracy of the discrimination.

## 2. Materials and Methods

### 2.1. Samples

Tomato samples were obtained from a nearby commercial farm in Beltsville, MD, USA. A total of 224 tomatoes were used, and were divided into two groups of 112 tomatoes regardless of coloration: one groups had cuticle cracks along the stem scar regions, and the other did not (sound). The samples were stored at room temperature (20 °C) for 24 h before hyperspectral imaging.

### 2.2. Hyperspectral NIR Imaging System

**Figure 1.**
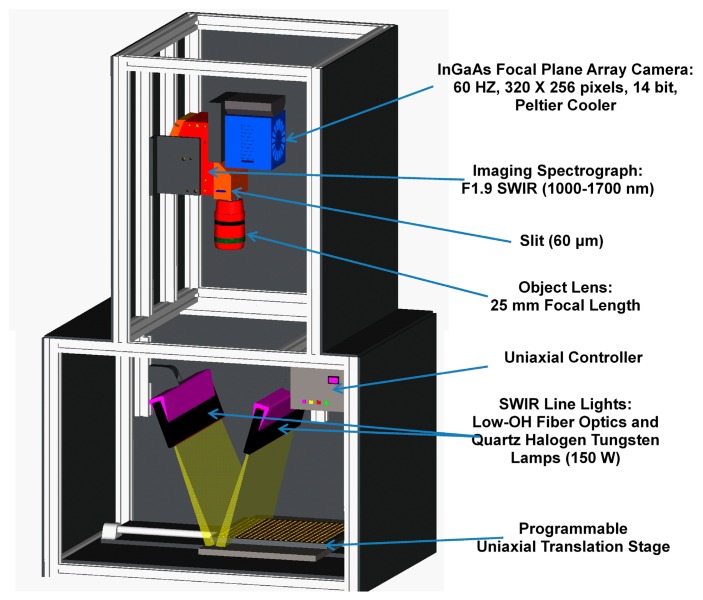
Schematic illustration of the critical components of the hyperspectral NIR imaging system.

A NIR hyperspectral imaging system was developed at the Environmental Microbiological and Food Safety Laboratory, Agricultural Research Service, USDA in Beltsville, MD, USA. Figure 1 illustrates the system and its critical components including a thermoelectrically (TE) cooled InGaAs camera (Xenics, Model XEVA-1.7-320, Leuven, Belgium) with a detector sized 320 × 256 pixels, a C-mount lens, line lights, and a programmable motorized sample stage. The effective spectral wavelength range of the system is 1000–1700 nm, with spectral intervals of approximately 4.8 nm (144 vertical pixels or channels). A pair of low-OH fiber optic line lights, each powered by a 150 W quartz tungsten halogen lamp (Dolan Jenner, Model DC-950, MA, USA), were positioned 15° forward and backward, respectively, from the vertical, and approximately 50 cm above the sample plate. A detailed description of the system and spectral calibration was reported previously [12].

### 2.3. Image Processing and Analyses

**Figure 2.**
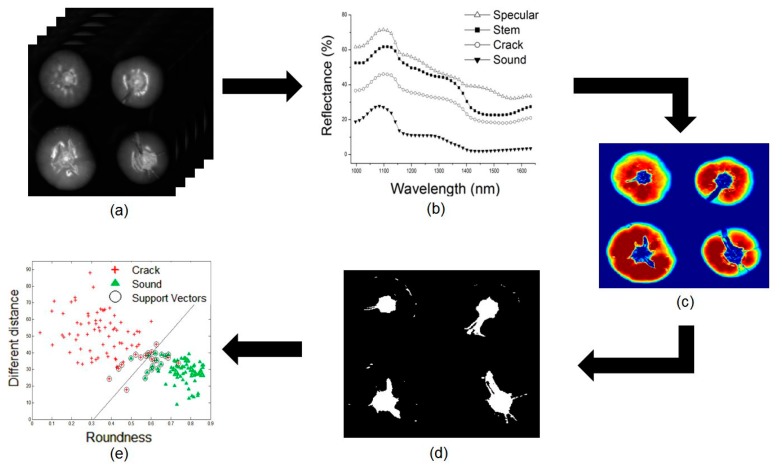
The procedure used to analyze cracked and sound tomatoes using hyperspectral NIR imaging. (**a**) Hyperspectral NIR imaging data were obtained. (**b**) Spectral analyses were then performed and a PLS-DA model was developed to detect cracks on the tomato. (**c**) A PLS-DA image, and (**d**) a PLS-DA binary image were obtained. (**e**) Finally, the data were classified using statistical methods.

Figure 2 shows the analytical procedure used for sample classification using hyperspectral NIR imaging data. First, the regions of interest (ROI) of the crack, stem, sound area, and a specular portion were extracted from the obtained hyperspectral NIR imaging data. Spectral analyses were conducted for each ROI and PLS-DA was used to detect cracked area from sound area. Feature values were obtained from the final images and then SVM and LDA were utilized to distinguish the two groups.

#### 2.3.1. Image Acquisition and Calibration

Four tomato samples were placed in the moving plate to measure at once and a total of fifty-six hyperspectral data were acquired. The exposure time and camera temperature (TE cooling) were set at 15 ms and 252 K, respectively. Four hundred twenty lines were gathered from individual samples and the hyperspectral imaging data consisted of three-dimensional 320 × 420 pixel and 144 waveband (channel) images. A white reflectance and dark current images were acquired to correct the obtained sample images. White reflectance images were acquired using a white PTFE reference panel (“Spectralon,” Labsphere, North Sutton, NH, USA) with approximately 99% reflectance, while dark reflectance images were obtained by covering the lens.

For acquiring the relative reflectance hyperspectral images of tomato samples, original hyperspectral image data were conducted by using following Equation (1).

(1) $$I=\frac{I_{0}-D}{W-D}$$

where *I* is the relative reflectance hyperspectral image, *I*_0_ is the original image data, *W* is the white reflectance image data, and *D* is the dark current image data.

#### 2.3.2. Spectra Extraction

The regions of interest for hyperspectral image were divided into four areas such as specular, stem, crack and sound. In order to extract ROI from the hyperspectral image manually, 1098 nm image presented highest intensity at hyperspectral image were used with the changing threshold value between 0.05 and 1. Of the two-hundred twenty four tomato samples, sixteen sound and cracked tomatoes were used to obtain representative spectra of each region.

#### 2.3.3. Partial Least Squares Discriminant Analysis

The spectrum obtained from each ROI was used to develop an optimal PLS-DA model, which can distinguish between cracked and sound tomatoes. PLS-DA is widely utilized for analyzing multi-dimensional data because of overcoming multi-collinearity and over-fitting. In addition, it can find latent variables by compressing large amounts of spectral data and describe the maximum covariance between cracked and sound spectra. It uses a multivariate least squares discrimination method, and so can be used when the independent variable is categorical. Equations of the PLS method are as following.

(2) $$X=TP^{T}+E$$

(3) $$Y=UC^{T}+F$$

(4) $$U=TB+G\left(B={\left(T^{T}T\right)}^{-1}T^{T}U\right)$$

where *X* is spectra data and *Y* is independent variables for two groups. After applying outer transform by orthogonal decomposition at *X* and *Y* matrices like (Equations (2) and (3)), construct inner relation between spectra data and independent variables by using least squares (Equations (4)) [15]. The optimal PLS-DA model was determined by minimum root mean square error of validation (RMSEV) using the following equation.

(5) $$\text{RMSEV}=\sqrt{\frac{\sum_{i=1}^{n}{\left(y_{v}-y_{\text{ref}}\right)}^{2}}{n}}$$

where *y_v_* is predicted value for developed model, *y_ref_* is independent variables, and *n* is the number of spectrum [16].

In the current study, the independent variables for two groups were set at 1 and 0, respectively. One thousand spectra from each region were selected randomly, and two different PLS-DA models were proposed; cracked *vs.* sound, and cracked *vs.* specular spectra. The beta coefficients for the developed model, which were derived from the suggested model, were used to create the PLS-DA image. A PLS-DA binary image was then obtained based on a threshold value of 0.5. The images were processed, such as by image filling followed by the labeling of connected components (by cluster size) and deleting the smaller clusters to leave only the largest cluster (determined by the labeling), to obtain an improved image and create binary images of the stem/cracked regions. All procedures were analyzed by using open function in MATLAB (version 7.0.4, Mathworks, Natick, MA, USA).

#### 2.3.4. Feature Extraction from PLS-DA Images

Since the cracks are mainly linked to the stems of the tomatoes, the values of two features, roundness (*R*) and differential distance (*D*), were calculated from the stem shapes on the images to classify the cracked and sound tomatoes. *R* was defined as “the bodily property of being well rounded in shape,” and was calculated using Equation (6). The closer *R* was to 1, the more circular the shape of the stem scar. *D* was calculated as the difference between the maximum away distance and minimum away distance from center of acquired stem scar image.

(6) $$\;\text{Roundness}\left(R\right)=\frac{4\times \pi \times \text{Area}}{{\text{Circumference}}^{2}}$$

#### 2.3.5. Classification Using Linear Discriminant Analysis and a Support Vector Machine

It is known that SVM and LDA, supervised learning method, are powerful tools that can distinguish two groups [17,18]. The basic theory of LDA is a method to find maximum value for ratio of between-classes scatter and within-class scatter, and SVM is to find maximum margin between two groups [19]. The linear discriminant equation and support vectors were derived from *R* and *D* values obtained from sound and cracked tomatoes and then were used to classify sound and cracked tomatoes. Seventy-five percent (n = 84) of the 112 tomato samples from each category (cracked and sound) were used as calibration data to develop the LDA and SVM models. The model derived from each calibration data set was then validated using an independent validation dataset (the remaining 25% [n = 28] tomato samples for each type of tomato). A commercial software package, MATLAB (version 7.0.4, Mathworks, Natick, MA, USA), was used for image processing and data analyses.

## 3. Results and Discussion

### 3.1. Spectral Analysis

**Figure 3.**
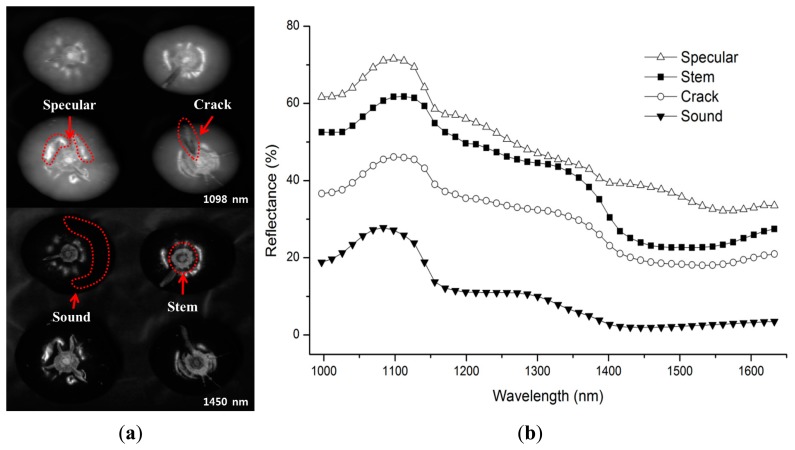
Representative reflectance images at 1098 nm and 1450 nm of tomatoes with cracks (**a**). Mean reflectance spectra of specular, stem-scar, cracks, and sound regions obtained from the hyperspectral NIR image data (**b**).

Figure 3a shows representative images of tomato samples obtained near the reflectance maximum (peak) and minimum (valley) at 1098 nm and 1450 nm, respectively. The relative reflectance spectra of the specular, crack, stem scar, and sound regions of tomatoes obtained in the spectral region between 1000 and 1700 nm are also shown in Figure 3b. The mean reflectance spectra were calculated from the pixel values of the ROIs in the reflectance images. The average reflectance obtained from cracked images was consistently higher than that obtained from the sound regions of the spectra. The decrease in reflectance was also associated with strong water absorption around 1190 nm and 1450 nm [20]. The intensity of the specular regions emanating due to surface and geometric properties of the tomato samples with respect to the illumination conditions was consistently higher than the reflectance intensity of other regions. The results showed that it is not possible to detect the crack areas on tomato with a single waveband. Hence, additional image processing steps were needed to detect cracks areas accurately without false-positive effects from the specular areas.

### 3.2. Imaging Processing Using PLS-DA

#### 3.2.1. Crack *vs.* Sound Regions

**Figure 4.**
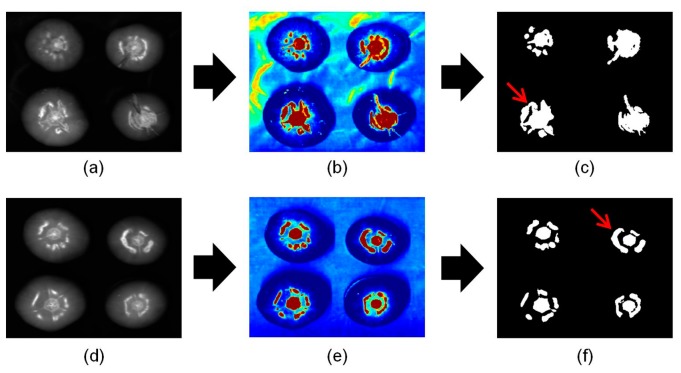
Imaging processing procedures by a PLS-DA model that could discriminate between crack and sound areas on tomatoes. (**a**) Single waveband image of a cracked tomatoes (1098 nm). (**b**) PLS-DA image of cracked tomatoes. (**c**) PLS-DA binary image of cracked tomatoes. (**d**) Single waveband image of sound tomatoes (1098 nm). (**e**) PLS-DA image of sound tomatoes. (**f**) PLS-DA binary image of sound tomatoes.

Lee *et al.* previously suggested that an algorithm and statistical methods such as analysis of variance (ANOVA) and principle component analysis could be used to detect cracks on tomatoes [21]. However, these methods were insufficient to detect only cracks from the resulting images because of the remaining specular image. Hence, imaging processing methods have been used to remove the specular regions. In the current study, we have developed a PLS-DA model, which was not suggested in the previous study to obtain enhanced images with reduced specular images. Figure 4 shows the sequence of events used to process images using the PLS-DA model, which can discriminate between cracked and sound areas on tomatoes. Figure 4b and 4e show PLS-DA images to which beta coefficients and hyperspectral NIR imaging were applied, respectively. Specifically, the PLS-DA image was separated into two parts: the crack, stem-scar, and specular part were close to red, and the sound part was close to blue. PLS-DA binary images obtained using a threshold value of 0.5 are shown in Figure 4c and f. Although PLS-DA was used to detect the cracked areas on tomatoes, the specular areas in final image were similarly emerged like previous research results. Therefore, the PLS-DA model obtained using spectra from the sound and cracked regions did not improve the results.

#### 3.2.2. Crack *vs.* Specular Regions

**Figure 5.**
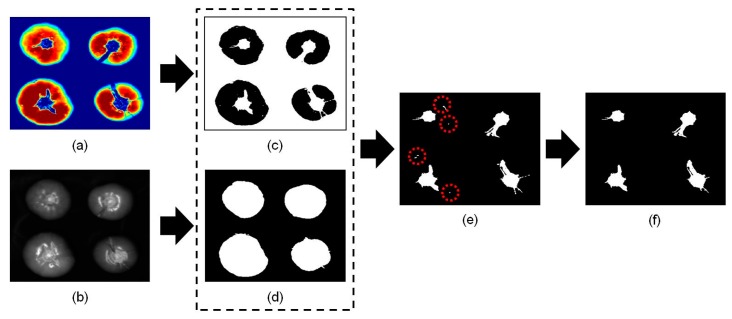
Image processing procedure to detect cracks on tomatoes. (**a**) PLS-DA image obtained from the re-developed model. (**b**) Single wavelength masking image obtained at 1098 nm. (**c**) PLS-DA binary image obtained using a threshold value of 0.5. (**d**) Masking image. (**e**) The image obtained by combining the PLS-DA binary image (c) with the masking image (d). (**f**) Final image after removal of the scattered pixels.

To overcome this problem and improve the data, the PLS-DA model was re-developed so that it detected only cracks on tomatoes using spectra of the specular and cracked areas. Figure 5 shows the sequence of image processing procedures used to detect cracks on tomatoes using the re-developed PLS-DA model. Figure 5a shows a PLS-DA image to which the re-developed model has been applied. In contrast to Figure 4a, the specular and sound regions were similar colors. In addition, the color of the crack and stem regions was comparable. Figure 5b shows a 1098 nm image; the masked image (Figure 5c) for the cracked tomato shown in Figure 5d was acquired by applying a 30% reflectance threshold value. The areas of stems with cracked regions could then be distinguished from the sound images. Figure 5e shows a combined image obtained from the PLS-DA binary image in Figure 5c and the masking image in Figure 5d. In Figure 5c, false positive areas with fewer than 20 pixels were deleted. The resulting image is shown in Figure 5f; it was then used to acquire the additional two features described below.

**Figure 6.**
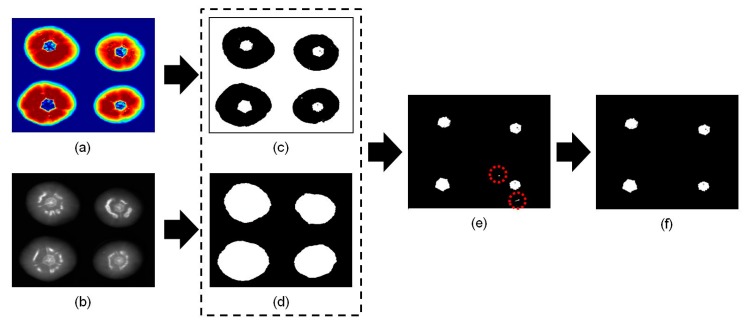
The sequence of events followed during image processing of the sound tomato. (**a**) Example of a PLS-DA image obtained using the re-developed model. (**b**) A single wavelength (1098 nm) masking image. (**c**) A PLS-DA binary image obtained using a threshold value of 0.5. (**d**) Masking image. (**e**) The image obtained by combining the PLS-DA binary image (c) with the masking image (d). (**f**) The final image obtained after removal of scattered pixels.

Figure 6 shows the sequence of processed images used to detect the stem scar on tomatoes. After applying a mask (Figure 6d) obtained from a 1098 nm image (Figure 6b), a PLS-DA binary image (Figure 6c) was obtained from the combination of the re-developed PLS-DA image (Figure 6a) and the masking image. False positive regions containing fewer than 20 pixels (marked by red circles on Figure 5e) were eliminated, resulting in a representative image of a sound tomato (Figure 6f). The final image in Figure 6f was then used in subsequent analyses to calculate the two features described below.

**Figure 7.**
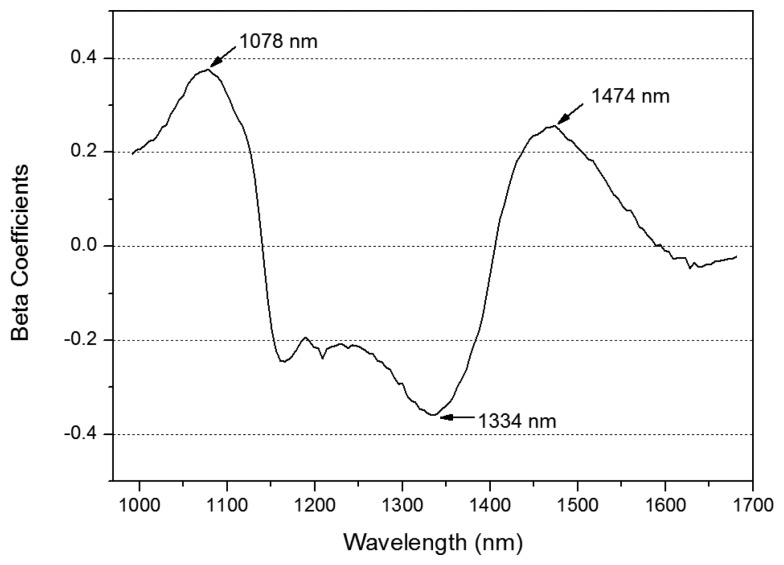
The beta coefficients of the re-developed PLS-DA model.

Figure 7 shows the beta coefficients for the re-developed PLS-DA model used to discriminate between cracked and sound tomatoes. Dominant peaks and valleys were observed at 1078 nm, 1334 nm, and 1474 nm. When a crack occurs on the surface of the tomato, wax (outermost epidermis) is destroyed whereby cutin was revealed. Then, cutin in cuticle cracks varied horny internal to protect plants from attack for outside such as contaminant water, fungi, and insects, and it was relatively dried condition compared with other regions [22]. The specular areas have importation of characteristic for sound area, as well as total reflection by surface materials and geometric properties between tomatoes and angle of light source. The prominent wavelengths at 1474 nm correspond to the second overtone of the O-H stretching mode related to water absorption, whereas the bands at 1078 nm and 1334 nm were not associated with any absorption component [23]. Although dominant chemical bands were not existed at 1078 nm and 1334 nm, two wavebands were statistically significant for classification of cracked tomatoes. It is likely that this was caused by differences in the chemical composition of the tissue (wax) and water content in sound and cracked areas, which allowed the cuticle cracks to be distinguished from the stem scar of the tomato.

### 3.3. Classification of Two Features Using LDA and SVM

**Figure 8.**
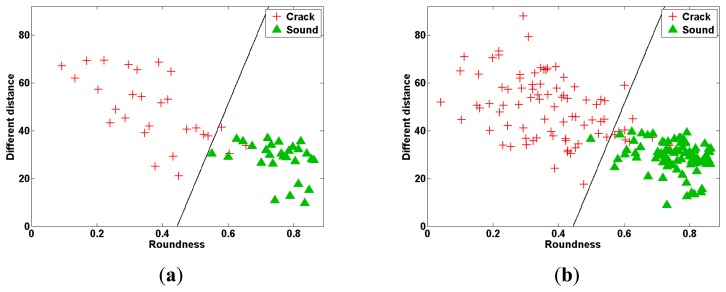
Classification of two features obtained from a PLS-DA binary image using LDA. (**a**) Calibration data set; (**b**) prediction data set.

**Figure 9.**
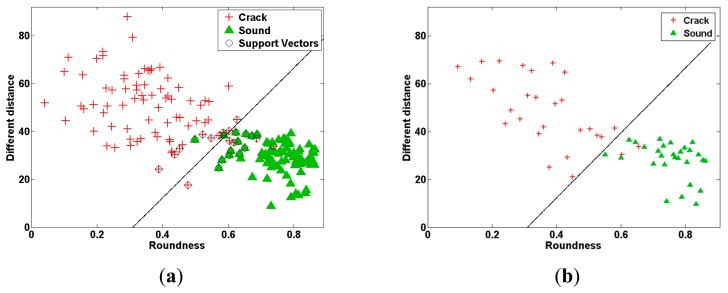
Classification of two features obtained from a PLS-DA binary image using SVM. (**a**) Calibration data set; (**b**) prediction data set.

The two stem crack features (*R* and *D*) calculated from the PLS-DA images of cracked and sound tomatoes were used to classify the two types of tomato. When only one feature (either *R* or *D*) was used to discriminate between tomato samples, the classification accuracy was ∼70%. LDA and SVM were proposed to improve the accuracy of classification between cracked and sound tomatoes. These techniques were used to obtain linear equations and support vectors for *R* and *D*, which were then used to classify the different types of tomato. Table 1 summarizes the classification accuracies obtained using the LDA and SVM methods in conjunction with the *R* and *D* values. The classification accuracy of the LDA-based method for identifying sound and cracked samples in the validation dataset was 100% and 89.3%, respectively, with an overall classification accuracy of 94.6%. The SVM-based *R* and *D* parameterization performed slightly better than the LDA method: it could separate sound and cracked tomatoes in the validation dataset with a classification accuracy of 100% and 92.9%, respectively. Scatter plots of the LDA- and SVM-based methods for the calibration and validation sets are shown in Figures 8 and 9, respectively. The total validation accuracy obtained using the SVM method was also higher than that from the LDA method (96.4% *vs.* 94.6%). These results are slightly higher than those of PCA and two-band ratio in previous study (PCA: 91.7 and Two-band ratio: 94.4%) [22].

If the outlier is present in the data of group, the variance of the data increases. Then, linear equation by outliers would be affected to differentiate the two groups because LDA method is based on the variance of the data. In contrast, SVM is made to be less affected by outliers because it is a method of using the optimal boundary margins of two groups. It is suggested that SVM is more appropriate method than LDA in this experiment.

## 4. Conclusions

Hyperspectral NIR images analyzed using a PLS-DA classification algorithm were used to detect cracks on tomatoes. Compared with previous studies, the PLS-DA method helps to obtain an enhanced image after the removal of the specular area, without the use of complicated image processing. Two morphological features, *R* and *D*, were calculated from binary images of tomato stem scars to identify and classify damaged tomatoes. Subsequently, SVM and LDA methods were used to assess *R* and *D* and classify cracked and sound tomatoes. The validation results obtained using the SVM and LDA methods revealed classification accuracies of 96.4% and 94.6%, respectively. Therefore, the PLS method and morphological parameterizations of stem scar features can be used to accurately discriminate between tomatoes with and without stem scar cracks. In addition, SVM method showed better performance than LDA method, and might be beneficial for inspecting cracked tomatoes.

## Figures and Tables

**Table 1.** Comparison of the results obtained using the SVM and LDA methods using two features obtained from the PLS binary images.

**Algorithm**		**Sound Samples (n = 112)**			**Cracked Samples (n = 112)**			**Total Accuracy (%)**

		**Correct**	**Incorrect**	**Accuracy (%)**	**Correct**	**Incorrect**	**Accuracy (%)**	
SVM	Calibration	82	2	97.6	79	5	94.0	95.8
	Validation	28	0	100	26	2	92.9	96.4
LDA	Calibration	81	1	98.8	76	8	90.5	94.6
	Validation	28	0	100	25	3	89.3	94.6
